# Seroprevalence of *Strongyloides stercoralis*, human T-lymphotropic virus, and Chagas disease in the Peruvian Amazon: a cross-sectional study

**DOI:** 10.1590/S1678-9946202466073

**Published:** 2024-12-16

**Authors:** Martin Casapía-Morales, Wilma-Selva Casanova-Rojas, Jhosephi Vázquez-Ascate, Cristiam-Armando Carey-Angeles, Carlos Alvarez-Antonio, Freddy-Franco Alava-Arévalo, Silvia Otero-Rodríguez, José-Manuel Ramos-Rincón

**Affiliations:** 1 Universidad Nacional de la Amazonia Peruana, Facultad de Medicina Humana, Iquitos, Peru; 2 Ministerio de Salud del Peru, Iquitos, Peru; 3 Hospital Universitario General Dr. Balmis, Departamento de Medicina Interna, Alicante, Spain; 4 Instituto de Investigación en Salud y Biomedicina, Alicante, Spain; 5 Universidad Miguel Hernández of Elche, Facultad de Medicina, Departamento de Medicina Clínica, Alicante, Spain

**Keywords:** Strongyloides stercoralis, HTLV, Chagas disease, Trypanosoma cruzi, Serology, Sero-prevalence, Prevalence, Peru, Amazon

## Abstract

*Strongyloides stercoralis* infections, human T-lymphotropic virus (HTLV) infections, and Chagas diseases occur throughout many regions of Central and South America, including Peru. This study aimed to evaluate the seroprevalence of *S. stercoralis*, HTLV, and Chagas disease in Iquitos (Peruvian Amazon) and the associated epidemiological conditions for *S. stercoralis* seroprevalence in Iquitos. A population-based cross-sectional study was conducted from May 1 to June 15, 2020, to assess the seroprevalence of *S. stercoralis* [lysate antigen ELISA (enzyme linked immunosorbent assay)], HTLV (recombinant antigen ELISA), and Chagas disease (crude and recombinant antigen ELISAs). Of the 396 included individuals, 257 were seropositive for *S. stercoralis* (a 64.9% prevalence, 95% confidence interval [CI] 60.0% to 69.4%). In the multivariable analysis, seropositivity for *S. stercoralis* was higher in women (odds ratio [OR] 1.60, 95% CI 1.03 to 2.66) and residents of Punchana (OR 3.47, 95% CI 1.51 to 7.93), whereas residence in Iquitos was associated with lower positivity (OR 0.52, 95% CI 0.32 to 0.85). In total, four individuals were positive for HTLV (1.0% seroprevalence, 95% CI 0.3% to 2.7%), and none were positive for Chagas disease (0.0% seroprevalence, 95% CI 0.0% to 1.2%). The seroprevalence of *S. stercoralis* in Iquitos is high, particularly among women and residents of Punchana. The presence of HTLV infection indicates that the virus is circulating in Iquitos. This study found no cases of Chagas disease.

## INTRODUCTION

Strongyloidiasis is an infection stemming from the human nematode parasite *Strongyloides stercoralis*. An estimated 370 million people worldwide have soil transmitted helminths (including *Ascaris lumbricoides*), *Trichuris trichiura*, hookworm, and strongyloidiasis. Strongyloidiasis is endemic in tropical and subtropical regions, as well as in some temperate areas, including Iquitos city in the Peruvian Amazon. The number of infected individuals and the risk of infection vary among population groups^1–3^. In Latin America, many countries have highly endemic areas^4,5^.

Various diagnostic methods can detect strongyloidiasis, including direct fecal smears, the Baermann technique, culture on Harada-Mori filter paper, charcoal cultures, and nutrient agar plate cultures. However, these methods have limited sensitivity^1–5^. Serological tests offer a useful tool in nonendemic areas due to their sensitivity^6,7^. Studies have analyzed the utility of serology to diagnose *S. stercoralis* in endemic countries, including in Latin America. Studies in Peru, Argentina, and Ecuador found that prevalence depends on the geographic area, the serological technique, and other factors^8–11^. Yori *et al*.^8^ reported a 72% prevalence in a rural community in the Peruvian Amazon, whereas Ortiz-Martínez *et al*.^9^ found a 33.7% prevalence among pregnant women in Iquitos city, also in the Peruvian Amazon. Similarly, Echazú *et al*.^10^ observed a 51 prevalence in a community in northwest Argentina and Anselmi *et al*.^11^ reported a 22.7% prevalence in a remote community in Ecuador. So, serological tests for *S. stercoralis* can serve as a valuable tool in endemic areas.

Of the two types of human t-lymphotropic virus (HTLV) — HTLV-1 and HTLV-2 —, HTLV-1 is associated with the development of tropical spastic paraparesis and adult T-cell leukemia/lymphoma^12,13^. In contrast, HTLV-2-associated diseases have received only occasional reports since it was first described^14^. HTLV infection is endemic in many regions of Central and South America and the Caribbean^12–14^. In Peru, HTLV infections occur throughout the country, the prevalence of which varies across regions^15^. A study of pregnant women in Iquitos city observed a seroprevalence of 1%^15^.

The parasitic protozoan *Trypanosoma cruzi* causes Chagas disease infection. It affects six to seven million people in Central and South America with a spectrum of clinical forms, including cardiomyopathy. This vector-borne disease (transmitted by triatomine bugs) can spread by maternal-fetal transmission^16^. Chagas disease is endemic to various areas of Peru, including the Greater Southern Region and northeastern departments of Cajamarca, Amazonas, San Martin, and Ucayali^17^. A recent study of Chagas disease in pregnant women in Iquitos city has reported a very low seroprevalence (0.33%)^18^.

This study aimed to evaluate the seroprevalence of *S. stercoralis* and the associated epidemiological conditions in Iquitos city. It also assessed the seroprevalence of HTLV and Chagas disease. This serological survey will help us understand the seroepidemiology of *S. stercoralis* (an intestinal nematode), Chagas disease (a protozoal disease), and HTLV (a retroviral infection) in Iquitos city and monitor the situation of these three diseases, which is somewhat neglected by public health authorities.

## MATERIALS AND METHODS

### Study design and population

A cross-sectional study in Iquitos city was conducted from May 1 to June 15, 2020. Iquitos city is the most densely populated municipality in the Peruvian Amazon as it houses 479,866 inhabitants (geographic location: 3°44’00"S 73°15’00"O). It is surrounded by three rivers (Amazon, Nanay, and Itaya) and Lake Moronacocha. The metropolitan Iquitos city is composed of four districts: Iquitos, Punchana, Belen, and San Juan Bautista^19^.

Participants were recruited for an epidemiological study of antibody prevalence against severe acute respiratory syndrome-related coronavirus 2 (SARS-CoV-2). A multi-stage, stratified sampling approach was employed, weighted by both gender and age groups, across the four aforementioned districts. It aimed to derive a representative sample of the population of Iquitos city. To achieve this, a four-level multi-stage sampling method was implemented, progressing from districts to blocks, households (defined as units sharing a kitchen or access to a common area), and finally to individuals. Based on an anticipated seroprevalence of 18%, a 95% confidence interval, and a 80% study power, the target sample size was calculated to total 725 participants. This estimation also accounted for an 80% eligibility rate and a 20% expected rejection rate. This study was approved by the Research Ethics Committee of the Loreto Regional Hospital (ID-053-CIEI-2020). All participants consented to sample collection and use of the sample for other seroepidemiological studies unrelated to SARS-CoV-2. All results were kept strictly confidential. Variables such as age, sex, district, occupation, and education level were collected, along with variables related to SARS-CoV-2 infection. Out of the 725 individuals initially enrolled in the SARS-CoV-2 study, 329 were excluded: 310 due to the absence of serum samples and 19 due to incomplete data.

### Sample analysis

Serum samples were aliquoted into 400-μL tubes and stored at −80 °C. The samples were thawed for serology testing. To detect immunoglobulin G (IgG) antibodies against *S. stercoralis*, the Strongyloides IgG IVD (in vitro diagnostic) ELISA (enzyme-linked immunosorbent assay) kit (DRG Instruments GmbH, Marburg, Germany), approved by the European Community, featuring microtiter wells coated with a soluble fraction of *S. stercoralis* L3 filariform larval antigen was used. The procedure was conducted according to the manufacturer's recommendations. A positive result was defined as 0.220 optical density (OD) units or greater and a negative result as less than 0.200 OD units, according to the manufacturer's instructions. Values from 0.200 to 0.219 OD units were considered indeterminate.

To assess the presence of antibodies against HTLV, ELISA (HTLV I+II ELISA recombinant v.4.0, Wiener lab, Rosario, Argentina) was used. Cut-off for being positive was negative control plus 0.200 OD according to the manufacturer's instructions^20^.

To detect T. cruzi IgG antibodies, two assays was used: the Chagatest ELISA lysate (Wiener, Rosario, Argentina) and the Chagatest ELISA recombinant v.4.0 (Wiener, Rosario, Argentina). *T. cruzi* infection was confirmed if both tests yielded positive results, whereas participants were considered seronegative if both tests yielded negative results. In case of discordance, a third serological test (immunochromatography) was performed. The Chagatests were completed according to the manufacturer's instructions, and the threshold for positive results was 0.100 OD units^21,22^.

### Data analysis

The collected data were systematically recorded and analyzed using IBM SPSS Statistics, version 26.0. Categorical variables were expressed as numbers and percentages. The risk factors for seropositivity to *S. stercoralis* were evaluated using the Chi-square test, with the results expressed as odds ratios (ORs) and 95% confidence intervals (CI). A multivariable logistic regression analysis was performed to identify independent risk factors for seropositivity to *S. stercoralis*. Variables with a P value below 0.1 in the univariate analyses and age were entered into a multivariate logistic regression model using a stepwise selection method with the likelihood ratio test. The CoxSnell R^2^ and the Nagelkerke R^2^ statistics were used to measure the strength of the relation between dependent (seropositivity to *S. stercoralis*) and independent variables.

## RESULTS

### Study population

Our sample included 396 individuals, of whom 53% were women and 62.5% were aged under 40 years. Participants had low comorbidity rates: 6.8% had hypertension; 3.5%, diabetes mellitus; 0.8%, chronic pulmonary disease; 0.3%, chronical renal insufficiency (creatinine clearance <45 mL/min); and no cases of severe liver disease, immunodeficiency, or severe neurological disease.

#### S. stercoralis serology

Of the 396 participants, 257 tested positives for *S. stercoralis*, evincing a prevalence of 64.9% (95% CI 60.0% to 69.4%). Table 1 shows the risk factors for seropositivity. Prevalence was higher in women than in men (70.0% vs 59.1%; P = 0.034) and varied among districts (San Juan Bautista, 66.2%; Belen, 71.7%; Iquitos, 50.0%; and Punchana, 88.2%). Risk factors in the multivariable analysis included the female sex (OR 1.60 95% CI 1.03 to 2.66) and residence in Punchana (OR 3.47, 95% CI 1.51 to 7.93), with a CoxSnell R^2^ statistic of 0.109 and a Nagelkerke R^2^ statistic of 0.150).

**Table 1:** Risk factors for positive serology against *Strongyloides stercoralis*.

		Total, % (n) (N = 396)	Serology result, % (n)		cOR (95% CI)	P	aOR (CI 95%)^a^	P
			Positive (N = 257)	Negative (N = 139)				
**Sex**								
	Male	47.0% (186)	59.1% (110)	40.9% (76)	1		1	
	Female	53.0% (210)	70.0% (147)	30% (63)	1.62 (1.06-2.44)	0.024	1.60 (1.03-2.66)	0.037
**Age (years)**								
	<19	23.5% (93)	64.5% (60)	35.5% (33)	1	-	1	
	20-39	38.4% (152)	67.8% (103)	32.2% (49)	1.15 (0.67-1.92)	0.600	1.02 (0.57-1.87)	0.931
	40-59	27.8% (110)	56.4% (62)	43.6% (48)	0.71 (0.40-1.23)	0.231	0.74 (0.40-1.32)	0.342
	≥ 60	10,4% (41)	78.0% (32)	22.0% (9)	1.95 (0.83-4.51)	0.123	2.05 (0.79-5.31)	0.137
**District**								
	S. J. Bautista	35.9% (142)	66.2% (94)	33.8 (48)	1^b^		1	
	Iquitos	35.4% (140)	50.0% (70)	50.0% (70)	0.51 (0.31-0.82)	0.006	0.52 (0.32-0.85)	0.010
	Punchana	17.2% (68)	88.2% (60)	11.8% (8)	3.83 (1.69-8.65)	0.001	3.47 (1.51-7.93)	0.003
	Belen	11.6% (46)	71.7 (33)	28.3% (13)	1.29 (0.62-2.69)	0.486	1.29 (0.61-2.72)	0.492
**Hypertension**								
	No	93.2 (369)	66.1% (244)	33.9% (125)	1	0.060	1	
	Yes	6.8% (27)	48.1% (13)	31.6% (14)	0.47 (0.21-1.04)		0.42 (0.17-1.04)	0.061
**DM**								
	No	96.5% (382)	65.2% (249)	34.8% (133)	1	0.576	—	
	Yes	3.5% (14)	57.1% (8)	42.9% (6)	0.71 (0.24-2.09)		—	
**Occupation**								
	Student	29.0% (115)	63.5% (73)	36.5% (42)	1		—	
	Self-employed	31.3% (124)	65.3% (81)	34.7% (43)	1.08 (0.641.84)	0.760	—	
	Salaried	39.6% (157)	65.5% (103)	34.4% (54)	1.09 (0.66-1.81)	0.717	—	

a Variables with a P value below 0.1 in the univariate analyses, along with age, were included in the multivariable logistic regression;

b The reference category was San Juan Bautista because it had the largest number of inhabitants included in this study; aOR = adjusted odds ratio; cOR = crude odds ratio; CI = confidence interval; DM = diabetes mellitus; n = number of participants to whom the variable applies; N = total number of participants in the group; S.J. Bautista = San Juan Bautista.

### HTLV and Chagas disease serology

In total, four participants tested positive for HTLV, evincing a seroprevalence of 1.0% (95% CI 0.3% to 2.7%). Table 2 summarizes the characteristics of seropositive individuals. No participants tested positive for Chagas disease (seroprevalence 0.0%, 95% CI 0.0% to 1.2%).

**Table 2:** Characteristics of four participants with human T-lymphotropic virus Infection.

		Total, % (n/N)
**Sex**		
	Male	0.5% (1/186)
	Female	1.4% (3/210)
**Age (years)**		
	< 19	0.0% (0/93)
	20-39	1.3% (2/152)
	40-59	0.9% (1/110)
	≥ 60	2.4% (1/41)
**District**		
	San Juan Bautista	0.7% (1/142)
	Iquitos	1.4% (2/140)
	Punchana	1.4% (1/68)
	Belen	0.0% (0/46)
**Hypertension**		
	No	1.1% (4/369)
	Yes	0.0% (0/27)
**Diabetes mellitus**		
	No	1.0% (4/382)
	Yes	0.0% (0/14)
**Occupation**		
	Student	0.0% (0/115)
	Self-employed	0.8% (1/124)
	Salaried	1.9% (3/157)
**Strongyloides stercoralis serology**		
	Positive	1.2% (3/257)
	Negative	0.7% (1/139)

n= number of participants to whom the variable applies; N = total number of participants to whom the variable applies.

#### *S. stercoralis* and HTLV serology

Form 257 individuals showing a positive *S. stercoralis* serology, two (1.2%; 95% CI: 0.5-5.9%)) had a positive HTLV serology, and from two individuals with a positive HTLV serology, three tested positive for *S. stercoralis* (prevalence 75%, 95% CI: 30.1-95.4%)

## DISCUSSION

The results of this study suggest that two out of every three people living in Iquitos city (Peruvian Amazon) have IgG antibodies against *S. stercoralis* and that the presence of antibodies is associated with the female sex and residing within the city. The rate of seropositivity was slightly lower than in Yori *et al*.^8^ with a native population on the Nanay river beside Iquitos city and lower than that our group found in pregnant women in Iquitos city^9^. We found no studies of seroprevalence in any region of Peru other than the Peruvian Amazon. Argentina shows an estimated prevalence of 50%^10,23^, whereas researchers in a rural area of Ecuador reported a prevalence of 20%^11^. Other seroepidemiological studies of *S. stercoralis* infection have shown prevalences ranging from 2.7% in Malaysia to 61.2% in Dhaka, the capital of Bangladesh^23–26^. The seropositivity in our sample more closely resembles that in Dhaka^26^.

This is an exploratory study of seroprevalence in Iquitos city. Exposure to contaminated freshwater and soil with barefoot offer a significant risk factor for *S. stercoralis* infection. The parasitic larvae can penetrate the skin, leading to infection^5^. The relation between *S. stercoralis* seropositivity and the female sex in this study may stem from their greater exposure to water. Women often engage in activities that increase their contact with soil and water^5^. Although some studies have found a higher prevalence in men^27,28^, the greater exposure to water in Iquitos city is due to the proximity of the rivers surrounding it and the consequent exposure to larvae in humid areas. Seroprevalence was highest in the Punchana district, at the confluence of two rivers (Nanay and Itaya), and lowest in the Iquitos district, in the central area of the city and with limited access to rivers.

Addressing this issue requires targeted public health interventions that focus on improving water quality and sanitation and education programs to raise awareness about the risks of *S. stercoralis* and other waterborne infections and the importance of protective measures, such as access to clean, safe drinking water; proper sanitation; hygiene; water treatment; and the wearing of shoes^5,28^. Understanding and mitigating the environmental and social factors contributing to *S. stercoralis* transmission can reduce the prevalence of this infection and improve the overall health of the community^29^.

The seroprevalence of HTLV in our population resembled that reported in previous studies in the area (1%)^15^. Mothers with HTLV infection offer a potential risk of mother-to-child transmission of infection during breastfeeding^30^. No participant was pregnant. Chronic HTLV infection can cause diseases such as tropical spastic paraparesis, T-cell leukemia, and severe *S. stercoralis* infection. When diagnosing these clinical forms associated with HTLV, clinicians should order HTLV serology to rule out coinfection. HTLV-1 infection may alter the immune response against *S. stercoralis* infection^31^. Individuals with HTLV infection and *S. stercoralis* co-infection may develop severe strongyloidiasis, which includes multiple organ manifestations, including pleuritis, pneumonia, and colitis^31^. In our study, 75% of patients with HTLV had positive serology for *S. stercoralis*, with an associated risk of severe strongyloidiasis.

No participants in our study were seropositive for Chagas disease, which supports previous findings that showed the low prevalence of this disease in the inhabitants of Iquitos city^18^. Chagas disease in the Peruvian Amazon has been mainly described as isolated cases and generally as acute disease^32^. In total, six acute Chagas disease cases in children from Indigenous Amazon communities in the province of Datem del Maranon (northeastern Peruvian Amazon, Loreto department) have been described, with a higher fatality rate (33%). Additionally, two species of adults triatomine, *Rhodnius pictipes* and *Rhodnius robustus*, were found inside the homes and in the peridomestic area^33^.

As this study recruited its participants for an epidemiological study of antibody prevalence to SARS-CoV-2, it now reviews the highlights of COVID-19 regarding *S. stercoralis* infection, HTLV, and Chagas disease. Regarding the impact of COVID-19 on *S. stercoralis* infection, patients with co-infection who were severely ill with COVID-19 and subjected to immunosuppressive treatment may develop severe *S. stercoralis* infection with acute respiratory distress syndrome^34^. These patients show different clinical characteristics than those without COVID-19, as per the finding that only 40% or less had fever or respiratory symptoms despite the severity of the *S. stercoralis* infection. Several reports detail cases of *S. stercoralis* hyperinfection with acute respiratory distress syndrome after treatment for COVID-19, some of which were fatal^34^. A systematic scoping review of the clinical features of *S. stercoralis* infection during the COVID-19 pandemic concluded that the potentially life-threatening and treatable nature of strongyloidiasis requires clinicians to recognize that patients with *S. stercoralis* infection in COVID-19 may show atypical features^34^. Therefore, routine rapid screening of critically ill COVID-19 patients undergoing immunosuppressive treatment and prompt anthelmintic treatment should be undertaken to prevent severe systemic *S. stercoralis* infection in at-risk patients^34^.

Regarding the association between COVID-19 and HTLV, no study has found a higher risk or mortality in cases of HTLV and COVID-19 co-infection. However, the most relevant finding is that patients living with HTLV who were vaccinated against COVID-19 showed significantly lower antibody activities^35,36^. This suggests a reduced humoral immune response to COVID-19 vaccines in this population^36,37^.

Regarding the association between COVID-19 and Chagas disease, several Brazilian studies have shown co-infection of Chagas disease and COVID-19 in Brazil in 2020, mainly in residents of Chagas disease-endemic areas. COVID-19 caused the highest percentage of deaths, significantly higher than those admitted due to other causes during the COVID-19 pandemic^38,39^.

Addressing parasitic diseases such as *S. stercoralis*, HTLV, and Chagas disease under the One Health approach is crucial due to the intricate interplay between human health, animal health, and the environment. For example, potential reservoirs of *S. stercoralis* in dogs and *Trypanosoma cruzi* in mammals, along with environmental factors such as climate change (rising temperatures, heavy rainfall, and storms), contribute to increase cases of *S. stercoralis* and Chagas disease. This comprehensive approach finds that the interconnected health of humans, animals, and ecosystems necessitate a collaborative effort across various disciplines. Studying these diseases via the One Health lens can better understand the multifaceted challenges posed by parasitic diseases, leading to improved health outcomes and a more resilient public health infrastructure^40^.

This study has successfully found and thoroughly analyzed various parasitic diseases by adjusting a population-based design for age and sex under a comprehensive One Health approach. This has enabled us to better understand the interconnectedness of human and environmental health. Our findings provide some points for developing effective prevention and control strategies for *S. stercoralis*, HTLV, and Chagas disease in Iquitos city.

However, this study has several limitations. First, its serological test may have cross-reactions with other helminthiases, such as ascariasis or hookworm infections. It would have been preferable to use an additional serological technique with different antigens to evaluate agreement with other serological tests. Second, it obtained no stool samples to verify the agreement of seropositivity with the identification of larvae in the stool. We consider this a non-serious limitation due to the low sensitivity of parasite identification in stools by non-molecular procedures. Third, as our sample was enrolled for a study to identify SARS-CoV-2 infection, the investigators neither collected variables associated with *S. stercoralis* infection, such as walking barefoot, bathing in rivers/streams, using municipal sewage, and other socioeconomic sanitary factors nor recorded symptoms of infection, such as diarrhea. Finally, regarding the seroprevalence of HTLV, the main limitation refers to the lack of a western blot test or INNO-LIA HTLV I/II essay to confirm the infection and differentiate HTLV-1 from HTLV-2. Thus, some of the four cases identified as HTLV-1/2 positive could be false positives, which suggests that the actual prevalence may be lower. Despite these limitations, our findings show the circulation of the virus in the city of Iquitos.

## CONCLUSION

In conclusion, the seroprevalence of *S. stercoralis* in Iquitos city is high and is related to the female sex and district of residence. The presence of HTLV infection in the inhabitants of Iquitos city highlights the potential risk of mother-to-child transmission during breastfeeding. On the other hand, Chagas disease is, rather than rare, absent in the city of Iquitos considering the representativeness of the survey. Chagas disease seems to be rare in the city of Iquitos. Further studies in other communities and based on other serological techniques are required to better understand the relevance of these high rates of seropositivity. The environmental and social factors contributing to *S. stercoralis* transmission should be studied. Moreover, prevalence studies of HTLV with identification of the circulating virus could provide valuable evidence.
